# The effect of antimicrobial photodynamic therapy on infected dentin in primary teeth: A randomized controlled clinical trial protocol

**DOI:** 10.1097/MD.0000000000015110

**Published:** 2019-04-12

**Authors:** Larissa Costa-Santos, Zenildo Santos Silva-Júnior, Ravana Angelini Sfalcin, Ana Carolina Costa da Mota, Anna Carolina Ratto Tempestini Horliana, Lara Jansinki Motta, Raquel Agnelli Mesquita-Ferrari, Kristianne Porta Santos Fernandes, Renato Araújo Prates, Daniela Fátima Teixeira Silva, Alessandro Deana, Sandra Kalil Bussadori

**Affiliations:** Nove de Julho University, São Paulo, State of São Paulo, Brazil.

**Keywords:** dental caries, low-level laser therapy, photodynamic therapy

## Abstract

**Background:**

Antimicrobial photodynamic therapy (aPDT) has been used for the treatment of dental caries. Papacarie is a gel composed of papain and chloramine employed for the partial removal of carious tissue, effective against bacteria, however, some studies report that this antibacterial action is not quite so evident. The aim of this study is to evaluate the clinical effect of aPDT on infected dentin in dental caries lesion in primary teeth.

**Methods:**

Thirty-two primary molars with deep occlusal dental caries will be selected and divided in 2 groups: G1 – caries removal with a low-speed drill and G2 – application of aPDT with PapacarieMBlue. After treatment, all the teeth will be restored with glass ionomer cement and followed up clinically and radiographically, with evaluations at 3, 6, and 12 months. Dentin samples before and after treatment will be analyzed microbiologically. The data will be submitted to descriptive statistical analysis of the association between the categorical variables and both age and gender using the chi-square test and Fisher exact text. The Student *t* test and analysis of variance will be used for the comparison of mean signs and symptoms of reversible pulpitis. Pearson correlation coefficients will be calculated for the analysis of correlations among the continuous variables.

**Discussion:**

Adding methylene blue dye to the formula of PapacarieMBlue might potentiate the antimicrobial action of aPDT and work more effectively on the infected dentin combined with a conservative, minimally invasive treatment.

**Trial registration:**

NCT02734420 on 10 march 2016.

## 1 Introduction

Dental caries remains a public health problem throughout the world and develops as the result of a dynamic process mediated by acid produced by cariogenic bacteria, leading to the loss of dental tissue.^[1]^ Minimal intervention combined with knowledge regarding the development of dental caries has led to major transformations in the restorative treatment paradigm, with a striking change involving the maximal preservation of sound dental tissue capable of remineralization.^[2,3]^

The partial removal of dental caries with the aim of maintaining the integrity of the pulp is currently considered the treatment of choice for deep carious lesions provided that certain diagnostic principles are respected.^[3–5]^ With minimally invasive clinical treatment using different procedures,^[6,7]^ the most superficial layer of infected dentin, which is irreversibly denatured and not capable of re-mineralization, is removed.^[5–8]^ The more internal or “affected” dentin is reversibly denatured and capable of remineralization and should be preserved.^[3,9–11]^ Thus, various aspects are important to the indication and performance of these procedures, such as the defense mechanism of the dentin-pulp complex in the occurrence of dental caries, the initial diagnosis, the differentiation of the types of dentin that compose the carious lesion, and the materials indicated for application to the remaining dentin.^[12–14]^

Papacarie^TM^ is a gel composed of papain and chloramines.^[3,15,16]^ The latter has disinfectant quality and the former is an enzyme similar to human pepsin that acts as a debriding agent that does not harm sound tissue and accelerates the healing process; papain also has bactericidal, bacteriostatic, and anti-inflammatory properties.^[17–19]^ Thus, it is associated with minimally painful treatment for caries lesions from primary teeth^[20,21]^ and exerts a positive result in bacterial reduction.^[21]^

Studies have demonstrated that antimicrobial photodynamic therapy (aPDT), have also been developed for the treatment of dental caries.^[22–27]^ The photodynamic action occurs when a photosensitizing agent absorbs photons from the light source and its electrons enter an excited singlet state.^[28]^ Different classes of chemical compounds, such as phenothiazines, phthalocyanines, and porphyrins, which have photoactive properties, have been effective in aPDT.^[23–26,28–32]^ Light must interact with the tissue in order to produce biological effect. The use of a chromophore applied to dentinal tissue has the function of absorbing light radiation, thereby increasing the interaction of the light with the tissue.^[22,23]^

Methylene blue (MB) is a widely known histological dye that has been in use for many years.^[33]^ It belongs to the phenothiazinium class of compounds. It is an amphiphatic tricyclic planar cationic molecule with 1 intrinsic quarternary nitrogen atom, and it presents phototoxic efficiency.^[34,35]^ Due to its photochemical and photobiological characteristics, widespread availability and ability to be excited with light, the aim of adding MB to the gel is to potentiate the antimicrobial action of aPDT.

## 2 Justification

Studies have shown that therapeutic measures, such as aPDT, have also been developed to the treatment of dental caries.^[22–26]^ However, there are few controlled clinical trials in the literature that confirm the effectiveness of this type of therapy on caries, especially in the primary dentition.

Controlled clinical trials developed to evaluate the clinical and antimicrobial action of Papacarie^TM^ have demonstrated its effectiveness against bacteria due to its properties.^[5,6,9]^ However, some studies report that this antibacterial action is not quite so evident. Thus, knowing the effective action of aPDT on infected dentin in young permanent teeth, as reported by Guglielmi et al in 2011,^[8]^ our aim in adding the dye to its formula was to develop a conservative treatment for the removal of carious tissue combined with the possible effective action against bacterial in infected dentin of primary teeth.

The justification for the addition of the dye for subsequent aPDT is to potentiate the antimicrobial action of aPDT and work more effectively on the infected dentin combined with a conservative, minimally invasive treatment.

## 3 Methods

### 3.1 Objectives

#### 3.1.1 General objectives

To investigate the use of aPDT on the infected dentin of primary teeth through a controlled clinical trial.

#### 3.1.2 Specific objectives

1. To compare photodynamic therapy with MB and PapacarieMBlue Papacarie^TM^ with regard to microbiological, radiographic, and clinical aspects.
2. To evaluate the antimicrobial effect of aPDT on infected dentin in primary teeth.
3. To perform a radiographic evaluation of the remaining dentin and radiographic density at 3 evaluation times over a 12-month period.

Hypothesis (H_1_): The administration of aPDT is effective in the treatment of infected carious dentin in primary teeth.

Hypothesis (H_0_): The administration of aPDT reduces the number of viable bacteria, but is not effective in the treatment of infected carious dentin in primary teeth.

#### 3.1.3 Trial design

Randomized, controlled, blind, clinical trial

#### 3.1.4 Participants

Volunteers: Thirty-two teeth will be selected from male and female children (with no restrictions regarding race or ethnicity) enrolled for treatment at the pediatric clinic of the dentistry course of Nove de Julho University (São Paulo, Brazil).

#### 3.1.5 Inclusion criteria

1. Adequate health with no systemic conditions;
2. Adequate cooperation;
3. Clinically presenting at least 1 primary molar with an acute, active caries on the dentin not surpassing 2/3 and only involving the occlusal face, with direct view and access as well as no clinical or radiographic signs of pulp involvement.

#### 3.1.6 Exclusion criteria

1. Systemic adverse health condition;
2. Uncooperative behavior;
3. Class II, III, IV, or V carious lesion based on Black classification;
4. Clinically: caries involving enamel, deficient restorations, small carious lesions on dentin with no access for manual scalers, hidden caries, sign or symptom of pulp involvement, clinical impossibility of restoration;
5. Radiographically: evidence of pulp involvement, carious lesion extending beyond 2/3 of dentin.

#### 3.1.7 Interventions

Group 1 – Caries removal with a low-speed drill.

1. Initial periapical and interproximal radiographs with double films;
2. Prophylaxis with toothbrush and fluoride toothpaste;
3. Relative isolation with lip bumper, cotton roll, and aspirator;
4. Microbiological sampling with ear curette for standardization of volume of carious tissue;
5. Removal of carious dentin with carbide burs and manual instruments;
6. Additional microbiological sampling;
7. Clinical inspection of texture of remaining dentin with an exploratory probe;
8. Restoration with glass ionomer cement (Ketac Molar EasyMix – 3M ESPE);
9. Clinical and radiographic follow-up.

Group 2 – Removal of carious tissue around cavity walls with dentin curette, administration of PapacarieMBlue modified with MB and aPDT.

1. Initial periapical and interproximal radiographs;
2. Relative isolation with lip bumper, cotton roll, and aspirator;
3. Microbiological sample with otoscope curette to standardize volume of carious tissue;
4. Application on PapacarieMBlue (addition of MB) for 5 minutes as a preirradiation time to potentiate effect of aPDT; removal of carious tissue around lateral walls of the cavity with noncutting curette; no removal of carious tissue on pulp floor;
5. Irradiation of dental tissue for 1 minute on a single point;
6. Second microbiological sample of remaining dentin with curette;
7. Clinical evaluation by inspection of texture of remaining dentin with exploratory probe;
8. Restoration with glass ionomer cement (Ketac Molar EasyMIx 3M ESPE);
9. Follow up;
10. Radiographic control: periapical and interproximal radiographs at 3, 6, and 12 months.

#### 3.1.8 Sample size

**Figure 1:**
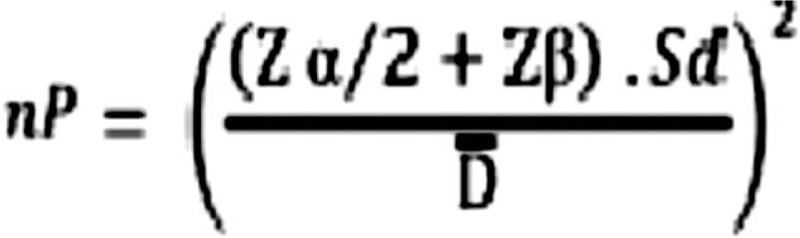
Sample size calculation used for this clinical research.

**Figure 2:**
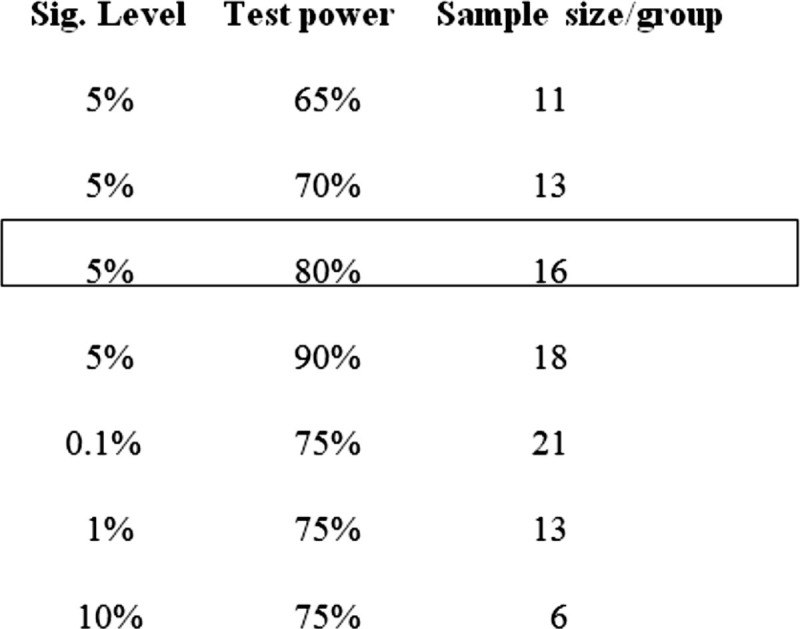
Determination of the sample size for this clinical research.

The sample size will be calculated based on a previous study in the literature^[30]^ (Fig. 1), considering the expected difference and standard deviation and weighting colony forming units (CFU) of bacteria. For the statistical calculation, paired samples were considered, with *α* = 1.96 (5%); *β* = 0.84 (20%), and an 80% test power. The minimum number in this clinical trial was determined to be 16 teeth per group (Fig. 2).

#### 3.1.9 Experimental groups

The experimental groups are described according to the Table 1.

**Table 1:**
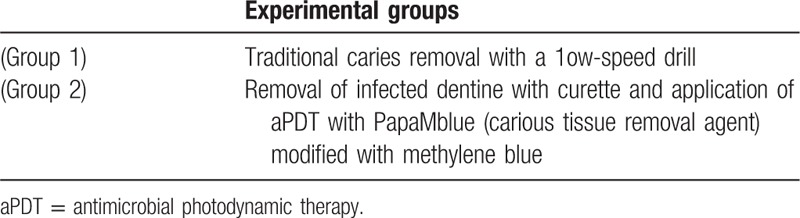
Experimental groups are described as shown below.

#### 3.1.10 Use of low-level laser for aPDT

For the use of the aPDT, the light source will be a low-level laser pen in the visible red band, a semiconductor (GaA1As and InGaA1P), The Laser DUO continuous mode, model from MM Optics, 100 mW, 2471/cm^2^, 148 J/cm^2^, irradiance 2471 mW/cm^2^. The device will be calibrated (*λ* = 660 nm, 6 J, 60 s), per spot.

The sessions will be held at the pediatric dentistry clinic. The volunteer and operator will be the only individuals present. Eye protection will be worn by both. The active tip of the laser will be covered with plastic wrap (PVC) for hygiene purposes and to avoid cross-contamination. The operator will be duly garbed. Based on a study by Guglielmi et al,^[8]^ the laser will be applied at a single point in contact with the cavity for each carious lesion. A conventional tip will be used and eye protection will be worn throughout the entire irradiation procedure to ensure the complete isolation of the patient's eyes.

As a randomized clinical trial, the recommendations of the Consolidated Standards of Reporting Trials^[36]^ will be used for greater transparency and quality.

#### 3.1.11 Procedures

Training and calibration of operator and examiner: All treatments will be performed by a single examiner who will undergo a training and calibration exercise in the initial phase of the study involving teeth randomly allocated to 2 groups. The operator will perform the treatment and a previously trained examiner will perform a clinical evaluation of the removal of carious tissue. The examiner will be unaware of the technique applied to the teeth (blind study). The previously trained examiner will evaluate all cavities after the respective interventions and will test the hardness of the remaining dentin. For carious tissue to be considered removed, there must be agreement between the operator and the examiner. This pilot study will be conducted to determine interexaminer agreement regarding the clinical and radiographic evaluations by examiners having undergone a calibration exercise with the calculation of Kappa coefficients. Interexaminer agreement must be greater than 85% (*K* > 0.85).

Randomization: The type of treatment will be determined based on the identification of the lottery stone:

Number 1 – Traditional caries removal with a low-speed drill (Group 1).

Number 2 – Removal of carious tissue around cavity walls with dentin curette, aPDT with PapacarieMBlue administration of PapacarieMBlue modified with MB followed by aPDT (Group 2).

Next, the individual and tooth will receive the previously determined treatment. Likewise, another tooth in the same individual will receive the treatment that was not determined previously.

Blinding: The clinical evaluations of the carious tissue removal, as well as the microbiological and radiographic analyses, will be performed by examiners blinded to the treatments performed on each tooth.

#### 3.1.12 Microbiological evaluation

A sample of infected dentin will be taken from each selected tooth before the removal of the carious tissue. The samples will be standardized with the use of a Meyhoefer auricular curette n° 2 and placed into test tubes containing 3.8 ml of transport medium (phosphate buffered saline; Labcenter, São Paulo, Brazil). The dental biofilm will be dispersed in the transport tube containing glass pearls through agitation at maximum speed in a vortex device for 30 seconds to homogenize the biological material. Next, the biofilm will be diluted in series on the order of 10^1^ to 10^6^ in peptone water and inoculated in culture media in Petri dishes. Aliquots of dilutions 10^4^, 10^5^, and 10^6^ will be sewn on the surface of Brucella agar (Difco, Kansas City) containing defibrinated sheep blood (50 ml/L), hemin (Inlab, São Paulo, Brazil) (5 mg/ml), and menadione (Inlab) (10 mg/ml) for the determination of the total number of viable microorganisms (VM). Aliquots of dilutions 10^3^ and 10^4^ will be sewn on Mitis Salivarius agar (Difco) for the determination of the total number of streptococcus (S). Aliquots of dilutions 10^1^ and 10^2^ will be sewn on Mitis Salivarius agar with the addition of debacitracin for the determination of the population of streptococcus of the mutans group (SM).^[34]^ Aliquots (100 μl) from each dilution will be sewn onto the surface of agar and spread with the aid of a Drigalski spatula. Undiluted aliquots and aliquots from dilution 10^2^ (100 μl) will be pour plated on Rogosa SL agar (Difco) for the determination of lactobacilli (LB)_._ The Brucella agar dishes will be incubated in an anaerobic chamber (PLAS by LABS, Lansing, MI) at 37°C for 7 days. The Mitis Salivarius agar and Mitis Salivarius Bacitracin dishes will be incubated in a 10% CO_2_ atmosphere (CO_2_ greenhouse, Shel Lab, mod. 2123, Oregon) at 37°C for 48 hours. The dishes containing Rogosa agar will be incubated in a 10% CO_2_ atmosphere at 37°C for 72 hours_._ After incubation, the characteristic colonies in each dish will be counted with the aid of a stereomicroscope at a magnification of 10 times in dilutions with 30 to 300 colonies per dish. All procedures will be performed in duplicate and the mean of the counts will be calculated. The results will be expressed in CFU of SM and LB as well as in proportion of streptococcus (% S/VM), SM group (% SM/VM and lactobacilli (% LB/VM) in relation to the total of VM. For SM, the proportion in relation to the total of streptococci (% SM/S) will also be calculated.^[37]^

Immediately after the removal of the carious tissue, samples of the remaining dentin will be taken with a Meyhoefer auricular n° 2 curette and the aforementioned procedures will be repeated.

#### 3.1.13 Radiographic evaluation

Periapical and interproximal radiographs will be taken initially and immediately after the procedure. Subsequently, follow up will be performed at 3, 6, and 12 months for the evaluation of optical density on the radiographs and the visual clinical interpretation of the remaining dentin as well as the evaluation using the radiographic subtraction method.^[38]^ All radiographs will be performed with double film (Kodak).

For a better understanding of the radiographic subtraction method, the 4 radiographs will be compared and taken with minimal geometric distortion, which is achieved by individualizing the premanufactured film positioners based on occlusal records using self-curing acrylic resin specific for each patient. The positioners will be stored for the subsequent radiographs. Exposure time, kilowatts and milliamperes of the device, type of film and radiographic processing will be standardized to obtain the most identical density and contrast possible among the radiograph.^[39]^ The radiographic subtraction methods in dentistry first began to study arterial vascularization of the mandible and are currently employed in different specialties.^[39]^ A positioner for interproximal radiographs will be used. The acrylic resin will be placed on the tooth surface and its antagonist for the anatomic impression of these surfaces and adapted to the positioner.^[40]^ This will enable the same position of the film at the different evaluation times and the standardization of the same x-ray incidence, vertical and horizontal angles and distance for all radiographs of the same patient.^[40]^

The same development time, intermediate rinsing, fixation and final rinsing will be standardized for all evaluation periods. The dark room temperature will be the same and Kodak chemicals will be used. The radiographic images from the different evaluation times will be scanned for the analysis of differences in density. For such, the Imagelab 2.3 program will be used.^[38]^

The density of the remaining dentin will be based on the changes in optical density. The teeth submitted to restorative treatment in both groups will be reevaluated after 3, 6, and 12 months. This method will consist of analyzing the standardized and digitized periapical and interproximal radiographs of 48 teeth in 48 patients aged 7 to 10 years to determine quantitatively the gray tones in the affected dentin region immediately below the restoration in class ionomer cement, the radiographic control of which for the visualization of sound dentin will allow the clinical examiner to compare the density of the remaining dentin in the different groups. The statistical analysis of optical density will be performed using the mixed-effects model.

#### 3.1.14 Evaluation of time required for procedure

The time required for each procedure will be measured using a digital stopwatch (Kenko, Hong Kong) in minutes and seconds from the onset of treatment until the complete removal of the carious tissue. The time will be recorded on a specific chart for analysis. The need or non-need for anesthesia will also be recorded.

#### 3.1.15 Evaluation of need for local anesthesia during intervention and degree of pain/discomfort of children during procedure

All interventions will be initiated without the prior administration of local anesthesia. The children will be told that anesthesia could be administered at any time during the intervention. A face scale with different expressions will be used to evaluate the need for local anesthesia and the child will be asked to point to the expression that most corresponds to his/her degree of pain/discomfort.

Interpretation of face scale:

1. No pain.
2. Mild pain.
3. Moderate pain.
4. A little worse pain.
5. Strong pain.
6. Worst pain.

#### 3.1.16 Clinical evaluation

The clinical evaluation will be performed by researcher blinded to the different treatment groups. The criteria used of the evaluation will be the retention of the restorative material in the cavity and the occurrence of secondary caries. The evaluation scores will be based on the results of previous studies.^[4]^ Digital photographs of the restorations will also be taken and serve to complement the clinical and radiographic findings. The visual demonstration will contribute to any necessary clarifications and facilitate the discussion and documentation of the cases. Thus, digital photographs will be taken (camera: Canon Sx500 IS) of all teeth in the different groups before and after the interventions.

0 = present; no defects;
1 = present; small marginal defects measuring less than 0.5 mm in depth; no need for repair;
2 = present; small marginal defects measuring 0.5 mm to 1 mm in depth; need for repair;
3 = present; large marginal defects measuring 1 or more mm in depth; need for repair;
4 = absent; restoration nearly or completely lost; need for treatment;
5 = absent; additional treatment having been performed for some reason;
6 = tooth absent for any reason;
7 = present; surface wear measuring less than 0.5 mm in depth; no need for replacement;
8 = present; surface wear greater than 0.5 mm in depth; need for replacement;
9 = impossible to diagnose.

#### 3.1.17 Statistical analysis

The data will be analyzed statistically employing different tests and considering a 5% level of significance. The results will be submitted to descriptive analysis for the association of categorical variables in age and gender using the chi-square test and Fisher exact test. The Student *t* test and analysis of variance will be used for the comparison of means of the signs and symptoms of reversible pulpitis. Pearson correlation coefficients will be calculated to determine correlations among the continuous variables.

## 4 Discussion

Therapeutic measures such as aPDT have also been developed to the treatment of dental caries,^[22–26]^ however, in the primary dentition, there are few controlled clinical trials in the literature confirming its efficacy. Papacarie^TM^ is a gel composed of papain and chloramine^[3,15,16]^ employed for the partial removal of carious tissue, effective against bacteria due to its properties, however, some studies report that this antibacterial action is not quite so evident. Adding MB dye to its formula might potentiate the antimicrobial action of aPDT and work more effectively on the infected dentin combined with a conservative, minimally invasive treatment. MB is a widely known histological dye that has been in use for many years.^[33]^ Due to its photochemical and photobiological characteristics, widespread availability and ability to be excited with light, the addition of the dye for subsequent aPDT is to potentiate the antimicrobial action of aPDT and work more effectively on the infected dentin combined with a conservative, minimally invasive treatment.

## Acknowledgments

This work was supported by the research grant FAPESP-Brazil 2016/11711-3. The authors gratefully thank Nove de Julho University for its technological support.

## Author contributions

LCS, ZSSJ, LJM, SKB participated in the conception and design of the study. LCS, ZSSJ, RAS, LJM, SKB participated in the data collection and drafting of the present manuscript. DFTS and AD performed statistical analysis. LJM and SKB critically reviewed the manuscript for intellectual content. ACRTH, RAMF, KPSF, RAP, and SKB coordinated the study. All authors read and approved the final manuscript.

**Conceptualization:** Larissa Costa-Santos, Zenildo Santos Silva-Júnior, Lara Jansinki Motta, Sandra Kalil Bussadori.

**Data curation:** Larissa Costa-Santos.

**Formal analysis:** Daniela Fatima Teixeira Silva, Alessandro Deana.

**Funding acquisition:** Larissa Costa-Santos, Renato Araújo Pratess.

**Investigation:** Sandra Kalil Bussadori.

**Methodology:** Larissa Costa-Santos, Ana Carolina Costa da Mota, Lara Jansinki Motta, Sandra Kalil Bussadori.

**Project administration:** Sandra Kalil Bussadori.

**Resources:** Larissa Costa-Santos.

**Software:** Renato Araújo Pratess, Daniela Fatima Teixeira Silva, Alessandro Deana.

**Supervision:** Ravana Angelini Sfalcin, Anna Carolina Ratto Tempestini Horliana, Lara Jansinki Motta, Raquel Agnelli Mesquita-Ferrari, Kristianne Porta Santos Fernandes, Renato Araújo Pratess, Sandra Kalil Bussadori.

**Validation:** Sandra Kalil Bussadori.

**Writing – original draft:** Larissa Costa-Santos, Ana Carolina Costa da Mota.

**Writing – review and editing:** Lara Jansinki Motta, Sandra Kalil Bussadori.
